# Comparative Effectiveness of Tocilizumab and TNF Inhibitors in Rheumatoid Arthritis Patients: Data from the Rheumatic Diseases Portuguese Register, Reuma.pt

**DOI:** 10.1155/2015/279890

**Published:** 2015-04-27

**Authors:** Vasco C. Romão, Maria José Santos, Joaquim Polido-Pereira, Cátia Duarte, Patrícia Nero, Cláudia Miguel, José António Costa, Miguel Bernardes, Fernando M. Pimentel-Santos, Filipe Barcelos, Lúcia Costa, José António Melo Gomes, José Alberto Pereira da Silva, Jaime Cunha Branco, José Canas da Silva, José António Pereira da Silva, João Eurico Fonseca, Helena Canhão

**Affiliations:** ^1^Rheumatology Research Unit, Instituto de Medicina Molecular, Faculdade de Medicina da Universidade de Lisboa, Lisbon Academic Medical Centre, Edifício Egas Moniz, Avenida Prof. Egas Moniz, 1649-028 Lisboa, Portugal; ^2^Rheumatology Department, Hospital de Santa Maria, Lisbon Academic Medical Centre, Avenida Prof. Egas Moniz, 1649-028 Lisboa, Portugal; ^3^Rheumatology Department, Hospital Garcia de Orta, Avenida Prof. Torrado da Silva, 2801-951 Almada, Portugal; ^4^Rheumatology Department, Centro Hospitalar Universitário de Coimbra, Praceta Mota Pinto, 3000-076 Coimbra, Portugal; ^5^Rheumatology Department, Hospital Egas Moniz, Centro Hospitalar Lisboa Ocidental, Rua da Junqueira 126, 1349-019 Lisboa, Portugal; ^6^Rheumatology Department, Instituto Português de Reumatologia, R. Beneficência 7, 1050-034 Lisboa, Portugal; ^7^Rheumatology Department, Unidade Local de Saúde do Alto Minho, Hospital Conde de Bertiandos, Largo Conde de Bertiandos, 4990-041 Ponte de Lima, Portugal; ^8^Rheumatology Department, Centro Hospitalar São João, Alameda Prof. Hernâni Monteiro, 4200-319 Porto, Portugal; ^9^CEDOC, NOVA Medical School, Nova University, Campo dos Mártires da Pátria 130, 1169-056 Lisboa, Portugal

## Abstract

*Objectives*. To compare the effectiveness of TNF inhibitors (TNFi) and tocilizumab in rheumatoid arthritis (RA) treatment, according to different response criteria. *Methods*. We included RA patients registered in the Rheumatic Diseases Portuguese Register treated with TNFi or tocilizumab for at least 6 months, between January 2008 and July 2013. We assessed remission/low disease activity (LDA) at 6 months according to DAS28, CDAI, and SDAI, as well as Boolean ACR/EULAR remission and EULAR response rate, adjusting for measured confounders. *Results*. Tocilizumab-treated patients (*n* = 95) presented higher baseline disease activity and were less frequently naïve to biologics compared to TNFi users (*n* = 429). Multivariate logistic regression analysis including the propensity score for receiving tocilizumab showed that patients treated with tocilizumab were more likely to achieve remission or LDA according to DAS28 (OR = 11.0/6.2, 95% CI 5.6–21.6/3.2–12.0), CDAI (OR = 2.8/2.6, 95% CI 1.2–6.5/1.3–5.5), or SDAI (OR = 3.6/2.5, 95% CI 1.5–8.7/1.1–5.5), as well as a good EULAR response (OR = 6.4, 95% CI 3.4–12.0). However, both groups did not differ in Boolean remission (OR = 1.9, 95% CI 0.8–4.8) or good/moderate EULAR response (OR = 1.8, 95% CI 0.8–4.5). *Conclusions*. Compared with TNFi, tocilizumab was associated with greater likelihood of achieving DAS28, CDAI, and SDAI remission/LDA and EULAR good response. Boolean remission and EULAR good/moderate response did not differ significantly between groups.

## 1. Introduction

Tumour necrosis factor inhibitors (TNFi) were the first biological agents introduced in the treatment of rheumatoid arthritis (RA). They have been widely used for over 15 years now and extensive evidence of their efficacy and effectiveness has accumulated, arising from numerous randomised clinical trials (RCTs) and large observational studies [1–6]. Tocilizumab, a monoclonal antibody targeting the interleukin-6 receptor, has become available one decade later and has progressively gained its place into RA treatment algorithms [7]. It has now been included in the last 2013 European League Against Rheumatism (EULAR) recommendations as one of the potential first line biologic drugs, alongside TNFi [7], after methotrexate (MTX) and/or other synthetic diseases modifying antirheumatic drugs (DMARDs) failure, a guidance followed by several national rheumatology societies [8].

TNFi are highly efficacious, both in monotherapy and in combination with synthetic DMARDs, such as MTX. Several indirect comparisons of RCTs and register-based observational studies failed to show significant differences in effectiveness between TNFi, although there are no available RCTs specifically addressing this issue [3–6, 9–12]. Likewise, tocilizumab presents good response rates, not only with concomitant MTX, but also in monotherapy [13, 14]. The only available head-to-head study, comparing tocilizumab and adalimumab in monotherapy, revealed higher clinical response with the former [15]. While RCTs directly assessing both classes of drugs in combination with synthetic DMARDs are missing, indirect comparisons through systematic reviews of RCTs have reported similar American College of Rheumatology (ACR) 50 responses [11, 12, 16–19] between tocilizumab and TNFi, with only one of these studies suggesting a higher ACR70 response rate with tocilizumab [16].

Real life observational data have confirmed the effectiveness of tocilizumab, with studies reporting 39–54.9% of the patients achieving remission according to disease activity score-28 joints (DAS28) [20–22] and 50.7% reaching ACR50 response at 24 weeks [22]. However, there are few observational register-based studies comparing the effectiveness of different biologic drug classes in real life circumstances. In one of such studies, Yoshida et al. compared the remission rates after 6 months of treatment with tocilizumab or TNFi and found that although the percentage of patients reaching DAS28-erythrocyte sedimentation rate (ESR) remission was higher with tocilizumab, the rates of stringent Boolean remission were similar in both groups [23]. This finding reflects the profound effect of tocilizumab upon inflammatory markers, due to the direct inhibition of IL-6, a major activator of the acute phase response [24]. Thus, response rates to tocilizumab might be overestimated when considering composite scores that include inflammatory markers, such as the DAS28, where ESR or C-reactive protein (CRP) has a high weight on the overall score [24].

With this in mind, we aimed to compare the effectiveness of TNFi and tocilizumab in RA treatment, according to different response criteria, in patients followed for at least 6 months in a multicentre nationwide cohort. We further looked at assessing the impact of previous biologic therapies on treatment response.

## 2. Methods

### 2.1. Patients

The Rheumatic Diseases Portuguese Register, Reuma.pt, is a nationwide clinical register established in 2008 and used in daily practice by nearly all rheumatology centers in Portugal [25]. Biologic therapy for RA has been available in Portugal since 2000, with the introduction of etanercept and infliximab. Adalimumab was approved in 2003 and the three drugs currently account for the majority of treatments. Tocilizumab and golimumab have become available in 2009 and 2010, respectively, and have progressively been incorporated into the daily clinical practice. The decision to initiate, switch, or maintain biologic treatment is guided by the SPR recommendations [8], which make no formal statement about which agent(s) should be considered as first line option(s).

We included patients fulfilling ACR 1987 revised RA criteria, starting tocilizumab or TNFi (adalimumab, etanercept, golimumab, or infliximab; certolizumab was not available in Portugal during the time frame of the study) between January 2008 and July 2013, who were treated for at least 6 months and had available DAS28 scores at baseline and follow-up.

All patients provided written informed consent as part of their enrolment in Reuma.pt, which is approved by competent authorities in Portugal, including the Health National Directorate and the National Board of Data Protection. The study was conducted according to the Declaration of Helsinki, as revised in Fortaleza (October 2013) and was approved by the Santa Maria Hospital Ethics Committee.

### 2.2. Statistical Methods

The coprimary outcomes were the proportion of patients in remission according to DAS28 (<2.6), CDAI (≤2.8), SDAI (≤3.3), and ACR/EULAR Boolean criteria (tender joint count-28 joints (TJC28) ≤ 1, swollen joint count-28 joints (SJC28) ≤ 1, CRP ≤ 1 mg/dL, and patient global health (PGH) ≤ 1/10). Secondary outcomes included proportion of patients reaching at least low disease activity (LDA) according to DAS28 (<3.2), CDAI (CDAI ≤ 10), and SDAI (≤11); frequencies of EULAR good (change in DAS28 > 1.2 and DAS28-6 months ≤ 3.2) and good/moderate response (change in DAS28 > 0.6 and DAS28-6 months ≤ 5.1 or change in DAS28 > 1.2 and DAS28 > 5.1); and, finally, change in DAS28, ESR, TJC28, SJC28, visual analogue scale (VAS), and health assessment questionnaire (HAQ). Covariates of interest included age at biologic start, gender, race (Caucasian versus non-Caucasian), disease duration, years of education, seropositivity (if anti-citrullinated protein antibody (ACPA) and/or rheumatoid factor (RF) positive), erosive disease (if erosions identified at X-rays of hands/feet at any time in disease), previous biologic therapy status (biologic-naïve versus previously exposed to ≥1 biologic), number of previous biologics, smoking status (current smokers versus noncurrent smokers), cardiovascular comorbidity (hypertension, dyslipidaemia, heart disease, or diabetes mellitus), and concomitant MTX and corticosteroid therapy. At 6 months, data were collected on TJC/SJC28, ESR, CRP, VAS (PGH and physician global assessment (PhGA)), and HAQ.

Baseline and follow-up data were compared according to biologic treatment using ANOVA, Student's *t*-test or chi-square tests, where applicable, both for each biologic separately and for biologic class (TNFi/tocilizumab). Bonferroni tests were applied, when significant differences were detected in ANOVA. We further performed stratification on previous biologic therapy status, to account for the potential relationship between previous biologic therapies and current therapy effectiveness.

To try to accommodate for patient- and disease-related confounders, we used multiple logistic regression and propensity score-based methods to explore the relationship between biologic class and treatment response. We built logistic regression models predicting binary response outcomes using stepwise backward elimination, including covariates with *P* value < 0.1 in the univariate analysis and those thought to be clinically meaningful (age, sex, seropositivity, number of previous biologics, disease duration, and baseline disease activity). In order to avoid overadjusting, individual components of the disease activity score were not considered. Variables conferring a greater than 10% change on the main regression coefficient (biologic class) were included in the final model.

A propensity score estimating the likelihood of receiving tocilizumab was generated, using a* logit* function and including baseline variables potentially related to biologic class that did not contain significant numbers of missing values: age, age-squared, sex, number of previous biologics, disease duration, baseline DAS28, TJC, SJC and concomitant treatment with MTX, corticosteroids, and other DMARDs. We then included this propensity score as a covariate in the univariate and multivariate logistic regressions in order to account for potential residual confounding. Finally, we conducted caliper 1 : 5 matching with replacement on the propensity score using the* psmatch2* command of Stata for each of the outcomes separately. Matching strategies significantly reduced the overall mean bias (e.g., 5.4% for the DAS28 matching), while decreasing the number of patients subject to the analysis, as expected.

All statistical analyses were performed using Stata version 12.1 (StataCorp, College Station, TX, USA) and *P* value was considered significant at <0.05.

## 3. Results

Five hundred and twenty-four patients fulfilled the inclusion criteria, 95 treated with tocilizumab and 429 with TNFi (106 adalimumab, 202 etanercept, 43 golimumab, and 78 infliximab). The baseline characteristics of the population are represented in Table 1. Patients from different groups had similar demographic characteristics, with expected distributions of variables such as age, gender, disease duration, smoking, or cardiovascular comorbidities, compatible with an established RA population. Frequencies of seropositivity (RF and/or ACPA), erosive disease and concomitant treatment with MTX, or low-dose corticosteroids were similar between groups considering either each biologic separately or biologic class. However, tocilizumab-treated patients were less frequently naïve to biologic therapy, had received a higher number of previous biologic agents, and had more active disease, as translated by significantly higher SJC28, PhGA, DAS28, CDAI, and SDAI. Furthermore, comparing patients by biologic class revealed higher mean ESR/CRP and increased proportions of patients with high disease activity according to all indexes in the tocilizumab group.

At follow-up (Table 2), only DAS28 and ESR were lower in the tocilizumab group compared to all TNFi (*P* < 0.001). Bonferroni tests after ANOVA regarding CRP at 6 months revealed that there were no significant differences between tocilizumab and each TNFi separately (*P* > 0.05 for all two-group comparisons). All other disease activity measures were similar between the groups. However, considering changes from baseline values, tocilizumab users presented a significantly greater decrease in DAS28, CDAI, SDAI, and inflammatory markers (ESR and CRP), as well as in the SJC28 and PhGA than patients treated with TNFi (Table 2).

### 3.1. Remission and EULAR Response

**Figure 1:**
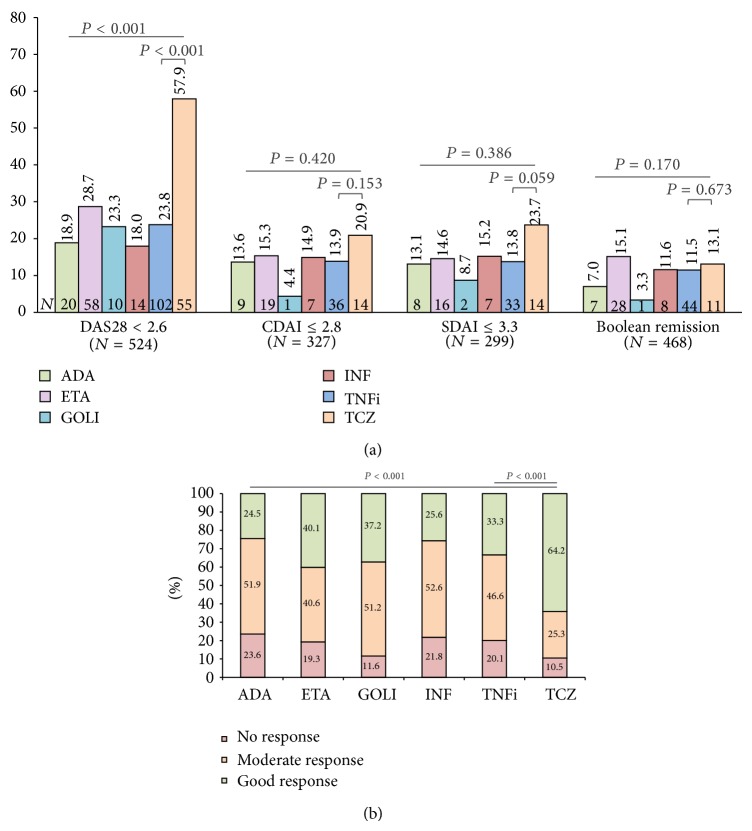
Frequencies of remission (a) and EULAR response (b) at 6 months according to biologic treatment. Tocilizumab- (TCZ-) treated patients had higher rates of DAS28 remission and EULAR response. Similar proportions of TCZ and TNFi users were in remission according to CDAI, SDAI, and Boolean remission criteria. *P* value significant at <0.05. ADA: adalimumab; ETA: etanercept; GOLI: golimumab; INF: infliximab.

More than half of tocilizumab-treated patients were in DAS28 remission at 6 months, a significantly higher proportion than observed for TNFi users (OR = 4.4, 95% confidence interval (CI) 2.8–7.0; Figure 1(a)). However, no significant differences were seen for remission rates according to CDAI (OR = 1.6, 95% CI 0.8–3.2), SDAI (OR = 1.9, 95% CI 0.97–3.9), or Boolean definition (OR = 1.1, 95% CI 0.6–2.3) criteria. Similarly to DAS28 change and remission, nearly two-thirds of the tocilizumab group had a good EULAR response, compared to one-third of TNFi users (OR = 3.6, 95% CI 2.3–5.7; Figure 1(b)). When considering the achievement of good/moderate EULAR response, the differences between groups were smaller, with 89.5% and 79.9% for tocilizumab and TNFi groups, respectively (OR = 2.1, 95% CI 1.07–4.2, *P* = 0.03).

### 3.2. Response according to Previous Exposure to Biologics

**Figure 2:**
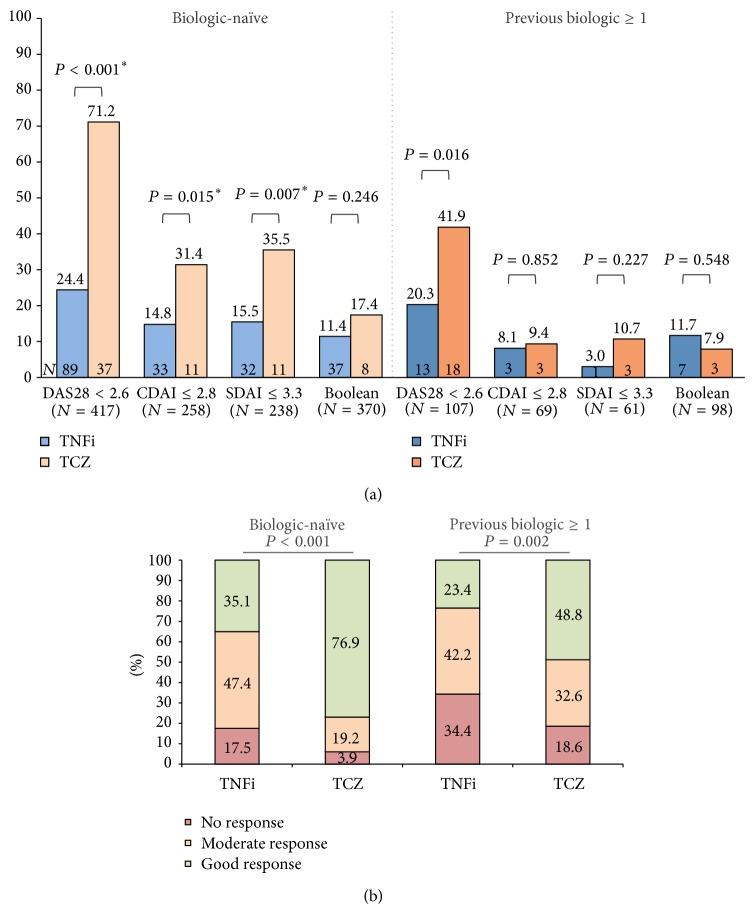
Remission (a) and EULAR response rate (b) at 6 months stratified by previous biologic therapy. (a) Biologic-naïve patients treated with TCZ had significantly higher rates of DAS28, CDAI, and SDAI remission, with nonsignificant differences in the more stringent Boolean remission. TCZ-treated patients previously exposed to at least 1 biologic had greater frequencies of DAS28 remission than those treated with TNFi, with similar rates of CDAI, SDAI, and Boolean remission. (b) EULAR response rates were significantly higher in the TCZ group for both previous biologic statuses, although the differences were greater in biologic-naïve patients. *P* value significant at <0.05.

Stratification according to previous biologic therapy exposure revealed that biologic-naïve patients treated with tocilizumab had higher odds of achieving remission, not only according to DAS28 (OR = 7.6, 95% CI 4.0–14.5), but also according to CDAI (OR = 2.6, 95% CI 1.2–5.8) and SDAI (OR = 3.0, 95% CI 1.3–6.8; Figure 2(a)). No significant differences were seen with the more stringent Boolean definition (OR = 1.6, 95% CI 0.7–3.7; Figure 2(a)). Regarding EULAR response, 76.9% of biologic-naïve patients in the tocilizumab group presented a good response, compared to 35.1% of TNFi users (OR = 6.2, 95% CI 3.2–12.1; Figure 2(b)). Considering patients previously exposed to biologic therapy and although the numbers are considerably smaller (TNFi = 64 and tocilizumab = 43), the results were similar to those observed in the overall population analysis, with significant differences being seen only for DAS28 remission (OR = 2.8, 95% CI 1.2–6.6; Figure 2(a)). Likewise, rates of EULAR response in this subgroup of patients were lower in comparison to those that were biologic-naïve, namely, 48.8% and 23.4% of EULAR good response in the tocilizumab and TNFi groups, respectively (OR = 3.1, 95% CI 1.4–7.1). Considering the achievement of at least a moderate EULAR response, differences between groups were less striking, being significant only in the biologic-naïve subgroup (OR = 5.3, 95% CI 1.3–22.4, *P* = 0.011) and not in patients previously exposed to biologics (OR = 2.3, 95% CI 0.9–5.7, *P* = 0.075).

### 3.3. Low Disease Activity at 6 Months

**Figure 3:**
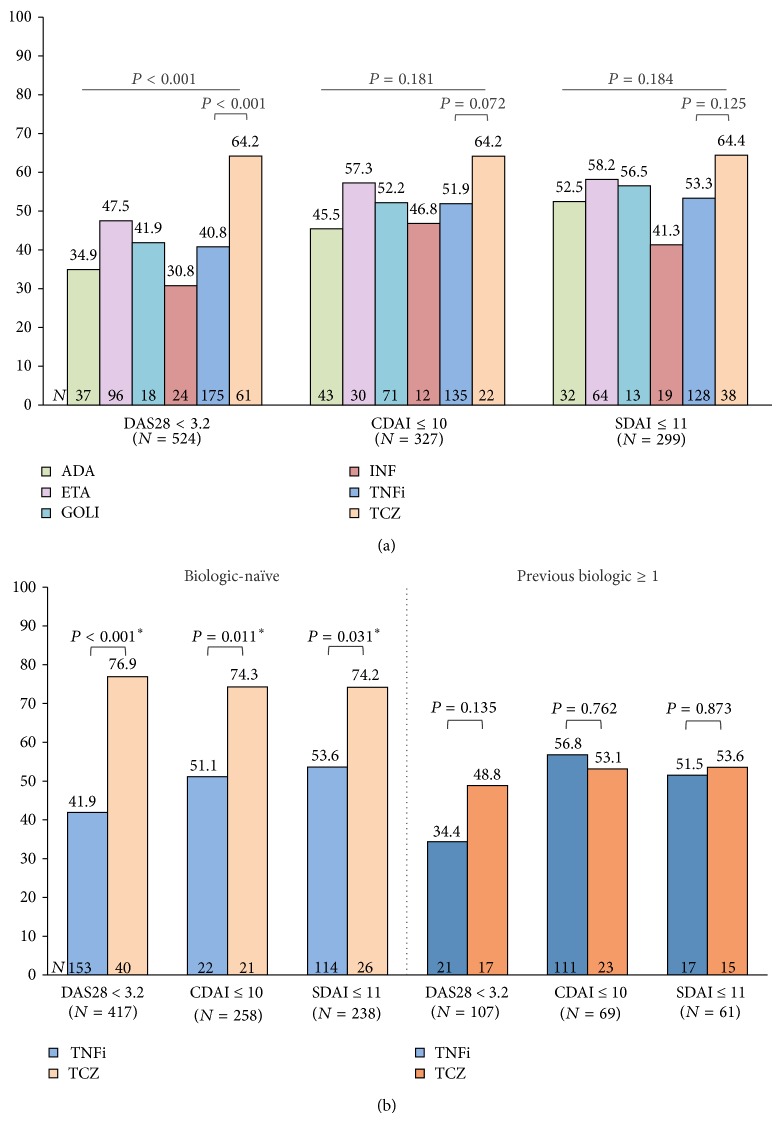
Low disease activity at 6 months according to treatment (a) and previous biologic therapy (b). (a) Significantly more patients treated with TCZ reached a state of at least DAS28 low disease activity (LDA), with no significant differences for CDAI and SDAI cutoffs. (b) Biologic-naïve patients in the TCZ group had significantly higher proportions of DAS28, CDAI, and SDAI LDA compared to TNFi users. On the contrary, in patients previously exposed to at least 1 biologic the frequencies of LDA according to all indexes were similar between drug class groups. *P* value significant at <0.05.

The proportions of patients achieving, at 6 months, at least a LDA state according to each of the indexes are represented in Figure 3 ((a) overall population analysis; (b) stratifying on previous biologic status). Notably, more than half of the patients reached LDA according to CDAI (≤10) or SDAI (≤11) regardless of type of treatment. As seen for remission, in the overall population analysis there were significant differences between groups favoring tocilizumab only for the DAS28 definition of LDA (OR = 2.6, 95% CI 1.6–4.1; Figure 3(a)). Interestingly, there was a better concordance between indexes than for remission, particularly in the tocilizumab group, with 64% of patients in LDA/remission according to every definition. Biologic-naïve patients also had higher odds of achieving at least a LDA state when treated with tocilizumab, compared to those in the TNFi group: defined by DAS28 (OR = 4.6, 95% CI 2.4–9.0), CDAI (OR = 2.8, 95% CI 1.3–6.1), or SDAI (OR = 2.5, 95% CI 1.1–5.7; Figure 3(b)). As for patients previously exposed to biologics, there were no statistically significant differences between both drug classes in terms of achieving at least LDA according to any of the criteria (Figure 3(b)).

### 3.4. Multivariate Analyses

Table 3 presents the results of logistic regression and propensity scores-based analyses to determine the effect size of tocilizumab versus TNFi in predicting each of the discussed outcomes. Multivariate logistic regression, adjusting for age, disease duration, baseline disease activity (DAS, CDAI, or SDAI, as appropriate), and number of previous biologics, revealed that tocilizumab-treated patients had higher odds of achieving remission and LDA according to DAS28, CDAI, and SDAI. There were no significant effects of biologic class on reaching Boolean remission. Good and good/moderate EULAR responses were also more likely to occur in tocilizumab-treated patients using this approach. The inclusion of the propensity scores predicting biologic class into the logistic regression model decreased the effect size of treatment group on the outcomes, although not changing the inference made for DAS28, CDAI or SDAI remission/LDA, Boolean remission, or EULAR good response. However, the odds of achieving a good/moderate EULAR response were no longer different between biologic therapy classes. Propensity score matching alone or in combination with multivariate logistic regression confirmed this finding and further revealed no significant effect of biologic class on reaching LDA according to SDAI. Achieving CDAI remission or LDA was not different between groups in the propensity score-matched analysis, although the regression analysis performed in the matched population did reveal significant differences for reaching remission/LDA, favoring TCZ. All other outcomes remained unchanged using this approach.

## 4. Discussion

In this study we compared the effectiveness of two classes of biologic therapies in RA patients registered in our national register, Reuma.pt. We found that patients treated with tocilizumab were more likely to achieve DAS28, CDAI, and SDAI remission/LDA, as well as good EULAR response at 6 months, when adjusting for confounding factors. On the other hand, the modelled probability of Boolean remission did not differ between groups and neither did the likelihood of achieving a good/moderate EULAR response when taking into account the propensity score for receiving tocilizumab.

Similar results were obtained by Yoshida et al., with fewer patients per group, in the single centre CABUKI register, where DAS28-ESR remission was more frequent in tocilizumab-treated patients (47.8% versus 25%, *P* = 0.006), Boolean remission was similar, and previous biologic therapy had a significant impact on DAS28 remission frequencies [23]. In another study and unlike us, Takahashi et al. found no significant differences in proportions of EULAR good response, DAS28-CRP remission, and LDA between patients treated with tocilizumab or adalimumab, while confirming higher frequencies of EULAR good/moderate response in the tocilizumab group [26]. No adjusted results were reported for these comparisons, an aspect that might help explain these discrepancies [26].

We have found an impressive frequency of 57.9% DAS28 remission at 6 months. These rates are higher than those observed in published RCTs (27% OPTION [27], 30.1% RADIATE [28], 39.9% ADACTA [15], and 40.4% ACT-RAY [13]) but similar to other observational register-based studies (39–58% DANBIO [20], 53.3% Michinoku Tocilizumab Study Group [29], 47.8% CABUKI [23], and 54.9% FRAB [21]). The EULAR response rates, based on DAS28, were also similar to those found in RCTs [13, 15, 27] and observational studies [20, 26] that reported them. While it is understandable that the remission rates seen in register-based studies are higher than those observed in RCTs, due to factors such as selection and attrition bias associated with observational studies in a real life setting, it is intriguing that such discrepancies are also seen among different registers. Although the magnitude of the difference in the proportion of patients achieving remission at 6 months is not very large (39% minimum in DANBIO [20], 57.9% maximum in Reuma.pt), several aspects can explain these findings. In observational studies, treatment is selected based on objective criteria and also on subjective physician attitudes and expectations, which greatly vary according to country or region. Western European practices certainly differ from Asian ones and even between Portugal (Reuma.pt) and Denmark (DANBIO) factors associated with treatment selection are quite different. Furthermore, visual analogue scale scores are highly subjective, influenced by local cultural factors, and can have a profound impact on the assessment of disease activity. Other potential explanations for these results include genetic factors accounting for variable responses between populations with distinct backgrounds and different frequencies of concomitant treatment with MTX, other DMARDs, and corticosteroids, which might be crucial for suppressing minimal disease activity and attaining remission.

The results of our study confirm that the inclusion of inflammatory markers in the assessment of response to therapy is of extreme importance when analyzing the effectiveness of drugs such as tocilizumab that, through a profound inhibition of IL-6-driven inflammation, markedly suppress ESR and CRP, even within the normal range limits, and might overestimate remission rates, as shown by Smolen and Aletaha [24]. These authors suggest the use of CDAI and SDAI remission/LDA might be more appropriate to compare treatment responses and, in fact, we have found no differences between biologic classes in the overall population analysis [24]. However, in biologic-naïve patients CDAI and SDAI remission were more frequent in the tocilizumab group, with OR of 2.6 (*P* = 0.015) and 3.0 (*P* = 0.007), respectively. Similar observations were made after multivariate analyses adjusting for several confounders. It should be noted, though, that the CDAI/SDAI-based analyses forced the exclusion of a significant number of patients due to missing values, and this might somewhat weaken the conclusions. However, sensitivity analysis revealed that simply excluding patients with missing values and performing a univariate analysis for the effect of biologic class on response rates did not yield the same results as the multivariate approaches (data not shown). This suggests that our results are not merely explained by the exclusion of patients in regression models.

We have also found that previous biologic therapy had an important effect on response to treatment. In fact, analysis of the biologic-naïve subgroup of patients revealed higher remission rates, especially for tocilizumab, not only according to DAS28, a finding already reported by others [21, 23], but also with CDAI and SDAI, which differed significantly between biologic class. While it seems reasonable that tocilizumab response is better in biologic-naïve patients compared to those having failed a biologic previously, the differences at 6 months between biologic class in terms of CDAI/SDAI remission/LDA might be at least partly explained by the fact that at baseline these subpopulations were more similar between groups, which was mainly due to less active disease in tocilizumab users and slightly more active disease in the TNFi group (data not shown; the only significant differences were SJC28 and DAS28, still both higher with tocilizumab). On the other hand, for TNFi users, response rates did not differ greatly between the biologic-naïve patients and both the overall population and those previously exposed to at least one biologic. This was also seen in the study by Yoshida et al. [23] and, in our opinion, might be related to two findings: first, most of the TNFi group (85.1%) was naïve to biologic therapy and, thus, the overall group mostly represented the characteristics of the biologic-naïve subpopulation; secondly, biologic-naïve TNFi users had higher baseline DAS28 (5.5 versus 5.1, *P* = 0.041) and SJC28 (7.1 versus 4.8, *P* < 0.001) compared to those that were previously exposed to biologic therapy, thus counteracting the potentially beneficial effect of being a first line user.

Our study has several limitations. Given its observational nature it is prone to different types of confounding, for which we have tried to account for by using propensity scores and multivariate logistic regression. However, residual and unmeasured confounding cannot be avoided and this may limit some of the conclusions. Furthermore, extrapolation of these results to drug efficacy is not possible. More specifically, the fact that there were several baseline differences between biologic class groups suggests that treatment was chosen, at least partially, based on the characteristics of the patients, as would be expected in a clinical practice setting. We used propensity scores to try to adjust for this, although we could not include every baseline variable of interest into the score due to missing data. However, given that RCTs addressing comparative effectiveness of biologics are unlikely to be conducted, observational studies are one of the ways to address this issue.

Other potential limitations should be also taken into account. In this study we provide data at 6 months, which might not be extendable to more prolonged follow-up times. We have also not analysed discontinuation rates or reasons, mainly due to missing values and short follow-up time, and this might limit the translation of conclusions into clinical practice. On the other hand, the fact that we have only included patients with available DAS28 at 0 and 6 months may also imply some degree of bias. Still, considering that Reuma.pt is a clinical practice register, data will be missing due to random reasons such as different likelihood of different clinicians to fill in all fields, rather than due to more or less severe disease activity. Another point to be focused is that we only included data on baseline concomitant MTX/corticosteroid treatment and did not assess whether these treatments were discontinued during follow-up. Nevertheless, given the relatively short follow-up and considering the longstanding nature of the disease in most cases, it is reasonable to assume that the combination therapy status did not alter significantly for the majority of the population.

## 5. Conclusions

In conclusion, using data from Reuma.pt, we found that treatment with tocilizumab was associated with higher rates of DAS28 remission/LDA and EULAR good response at 6 months. Similar observations were made also for CDAI and SDAI remission in biologic-naïve patients. Adjusting for other potential confounders confirmed higher response rates with tocilizumab according to DAS28, CDAI, and SDAI criteria. Boolean remission was similar between groups, suggesting that the use of more stringent remission criteria blurs the differences between drug classes. Similar, larger and longer observational studies from other registers are needed to confirm these results and give further insight into therapeutic decisions in the managements of RA patients.

## Figures and Tables

**Table 1:** Baseline characteristics of included rheumatoid arthritis patients.

	Adalimumab (*n* = 106)	Etanercept (*n* = 202)	Golimumab (*n* = 43)	Infliximab (*n* = 78)	Tocilizumab (*n* = 95)	*P* value^§^	TNFi (*n* = 429)	*P* value^¥^
Age (years)	52 ± 11.0	53.1 ± 13.3	55.2 ± 11.4	54.9 ± 11.9	53.8 ± 10.9	0.558	53.5 ± 12.3	0.791
Female	98 (92.5)	172 (85.2)	38 (88.4)	67 (85.9)	82 (86.3)	0.459	375 (87.4)	0.772
Caucasian (*n* = 456)	85 (92.4)	166 (95.4)	27 (96.4)	67 (89.3)	80 (92.0)	0.424	345 (93.5)	0.607
Disease duration (years, *n* = 489)	12.3 ± 10.0	11.1 ± 9.0	10.2 ± 8.5	13.1 ± 10.6	10.7 ± 9.0	0.339	11.7 ± 9.5	0.372
Education (years, *n* = 387)	7.2 ± 4.7	7.4 ± 4.7	7.5 ± 3.6	6.2 ± 4.1	7.4 ± 4.6	0.464	7.1 ± 4.5	0.611
Current smokers (*n* = 450)	11 (11.6)	23 (13.0)	2 (8.0)	7 (10.1)	12 (14.3)	0.884	43 (11.8)	0.522
CV comorbidity (*n* = 467)	50 (52.1)	68 (39.5)	14 (36.8)	28 (38.9)	40 (44.9)	0.258	160 (42.3)	0.654
Seropositive (*n* = 463)	80 (87.0)	142 (80.2)	29 (76.3)	61 (92.4)	73 (81.1)	0.107	312 (83.7)	0.564
Erosive (*n* = 380)	18 (25.4)	37 (23.7)	7 (25.9)	13 (23.6)	16 (22.5)	0.994	75 (24.3)	0.757
Previous biologics	0.24 ± 0.61	0.16 ± 0.38	0.09 ± 0.29	0.14 ± 0.39	0.81 ± 1.13	**<0.001**	0.17 ± 0.44	**<0.001**
Biologic-naïve	88 (83.0)	170 (84.2)	39 (90.7)	68 (87.2)	52 (54.7)	**<0.001**	365 (85.1)	**<0.001**
MTX	86 (81.1)	164 (81.2)	36 (83.7)	67 (85.9)	75 (79.0)	0.813	353 (82.3)	0.447
MTX dose (mg/week)	19.6 ± 4.4	18.9 ± 4.5	19.4 ± 5.2	19.6 ± 3.8	18.2 ± 4.2	0.279	19.3 ± 4.4	0.069
Corticosteroids	81 (76.4)	153 (75.7)	35 (81.4)	65 (83.3)	77 (81.1)	0.586	334 (77.9)	0.493
Corticosteroids dose (mg/day)	7.4 ± 3.3	7.3 ± 2.9	7.2 ± 2.8	7.1 ± 2.7	6.7 ± 2.4	0.530	7.3 ± 3.0	0.097
TJC28	11.1 ± 8.2	10.1 ± 7.3	9.2 ± 6.8	11.3 ± 8.2	12.4 ± 7.5	0.092	10.5 ± 7.6	**0.028**
SJC28	7.0 ± 5.5	6.5 ± 4.7	6.9 ± 4.6	7.2 ± 5.7	10.4 ± 6.4	**<0.001**	6.8 ± 5.1	**<0.001**
ESR (mm/h, *n* = 522)	36.2 ± 22.9	36.9 ± 27.2	38.9 ± 27.1	37.7 ± 24.4	45.6 ± 27.1	0.073	37.1 ± 25.6	**0.004**
CRP (mg/dL, *n* = 491)	2.2 ± 2.6	2.0 ± 3.1	2.2 ± 2.7	1.9 ± 1.9	2.8 ± 3.2	0.266	2.1 ± 2.7	**0.035**
PGH (mm, *n* = 496)	58.7 ± 24.5	56.4 ± 22.9	59.5 ± 20.2	60.5 ± 23.6	59.8 ± 24.3	0.648	58.0 ± 23.2	0.496
PhGA (mm, *n* = 376)	47.3 ± 20.1	51.5 ± 20.0	51.0 ± 19.1	54.4 ± 19.2	60.0 ± 17.9	**0.002**	51.0 ± 19.8	**0.001**
DAS28	5.5 ± 1.4	5.4 ± 1.3	5.4 ± 1.2	5.6 ± 1.4	6.1 ± 1.1	**0.001**	5.4 ± 1.3	**<0.001**
CDAI (*n* = 376)	27.7 ± 14.8	28.0 ± 12.8	26.0 ± 11.5	29.8 ± 14.9	33.3 ± 13.2	**0.037**	28.1 ± 13.6	**0.003**
SDAI (*n* = 361)	29.9 ± 15.4	30.6 ± 13.8	27.6 ± 12.0	31.7 ± 15.7	35.6 ± 13.1	0.056	30.4 ± 14.4	**0.006**
HAQ (*n* = 415)	1.6 ± 0.7	1.4 ± 0.6	1.5 ± 0.7	1.5 ± 0.6	1.6 ± 0.6	0.158	1.5 ± 0.6	0.150
High disease activity								
DAS28 (>5.1)	68 (64.2)	120 (59.4)	28 (65.1)	51 (65.4)	74 (77.9)	**0.044**	267 (62.2)	**0.004**
CDAI (>22, *n* = 376)	46 (60.5)	93 (65.0)	14 (51.9)	38 (64.4)	56 (78.9)	0.068	191 (62.6)	**0.009**
SDAI (>26, *n* = 361)	39 (54.2)	78 (58.7)	13 (48.2)	33 (55.9)	53 (75.7)	**0.036**	163 (56.0)	**0.003**

Continuous variables presented as mean ± standard deviation; categorical variables are expressed as number (percentage). Final number of patients is indicated where data was missing. *P* value significant at <0.05; significant differences highlighted in bold. ^§^Comparison of groups according to biologic using ANOVA or chi-square test, as appropriate; ^*¥*^comparison of TNFi versus tocilizumab groups using Student's *t*-test or chi-square test, as appropriate. CDAI: clinical disease activity index; CRP: C-reactive protein; CV: cardiovascular; DAS28: disease activity score-28 joints; ESR: erythrocyte sedimentation rate; HAQ: health assessment questionnaire; MTX: methotrexate; PGH: patient global health; PhGA: physician global assessment; SDAI: simplified disease activity index; SJC28: swollen joint count-28 joints; TJC28: tender joint count-28 joints; TNFi: tumour necrosis factor inhibitors.

**Table 2:** Disease activity at 6-month follow-up and respective change from baseline.

	Adalimumab (*n* = 106)	Etanercept (*n* = 202)	Golimumab (*n* = 43)	Infliximab (*n* = 78)	Tocilizumab (*n* = 95)	*P* value^§^	TNFi (*n* = 429)	*P* value^*¥*^
DAS28 ΔDAS28	3.8 ± 1.4 1.6 ± 1.4	3.5 ± 1.4 1.9 ± 1.4	3.5 ± 1.2 1.9 ± 1.4	3.8 ± 1.4 1.8 ± 1.4	2.8 ± 1.6 3.3 ± 1.6	**<0.001** **<0.001**	3.6 ± 1.4 1.8 ± 1.4	**<0.001** **<0.001**

CDAI (*n* = 327) ΔCDAI	12.4 ± 9.3 15.1 ± 12.5	11.7 ± 10.0 16.1 ± 14.8	10.6 ± 6.5 16.0 ± 8.5	12.7 ± 10.2 17.0 ± 14.3	10.5 ± 10.3 22.7 ± 15.7	0.690 **0.017**	12.0 ± 9.6 16.0 ± 13.6	0.254 **0.001**

SDAI (*n* = 299) ΔSDAI	13.7 ± 11.1 16.1 ± 13.3	12.4 ± 10.5 17.6 ± 16.1	13.1 ± 13.0 15.2 ± 11.6	14.0 ± 10.7 18.2 ± 14.9	11.6 ± 11.5 25.2 ± 16.5	0.778 **0.007**	13.1 ± 10.9 17.1 ± 14.7	0.343 **0.0003**

TJC28 ΔTJC28	4.8 ± 5.8 6.3 ± 6.9	3.7 ± 5.4 6.5 ± 7.5	2.7 ± 3.2 6.5 ± 6.3	4.6 ± 5.9 6.6 ± 7.5	3.6 (4.8) 8.7 ± 7.7	0.109 0.100	4.0 ± 5.5 6.4 ± 7.2	0.508 **0.006**

SJC28 ΔSJC28	2.3 ± 3.0 4.6 ± 4.8	1.7 ± 2.6 4.7 ± 4.9	2.3 ± 2.9 4.6 ± 4.4	2.2 ± 3.2 5.0 ± 4.9	2.6 ± 3.9 7.8 ± 6.6	0.179 **<0.001**	2.0 ± 2.9 4.7 ± 4.8	0.111 **<0.001**

ESR (mm/h, *n* = 516) ΔESR	26.2 ± 21.6 10.1 ± 21.2	23.7 ± 19.4 13.5 ± 22.5	22.1 ± 15.1 15.5 ± 21.9	25.9 ± 21.0 12.0 ± 23.0	10.3 ± 14.5 35.2 ± 25.1	**<0.001** **<0.001**	24.6 ± 19.9 12.6 ± 22.2	**<0.001** **<0.001**

CRP (mg/dL, *n* = 465) ΔCRP	1.4 ± 2.7 0.7 ± 3.0	0.81 ± 1.4 1.2 ± 3.1	1.9 ± 7.3 0.1 ± 7.5	1.0 ± 1.7 1.0 ± 1.7	0.5 ± 1.3 2.3 ± 3.2	**0.023** **0.006**	1.1 ± 2.9 0.9 ± 3.5	**0.045** **0.0009**

PGH (mm, *n* = 481) ΔPGH	39.8 ± 25.8 18.7 ± 31.6	34.9 ± 23.8 21.5 ± 26.4	41.7 ± 22.0 18.3 ± 19.9	38.9 ± 24.9 21.5 ± 28.9	35.0 ± 24.0 25.5 ± 30.0	0.295 0.525	37.4 ± 24.4 20.5 ± 27.7	0.385 0.132

PhGA (mm, *n* = 327) ΔPhGA	25.8 ± 17.3 20.5 ± 21.4	24.0 ± 17.9 26.7 ± 23.2	22.7 ± 14.7 29.1 ± 19.1	28.3 ± 20.4 27.4 ± 21.6	20.0 ± 19.4 39.8 ± 25.6	0.160 **0.0001**	25.1 ± 18.0 25.5 ± 22.2	**0.041** **<0.001**

HAQ (*n* = 323) ΔHAQ	1.1 ± 0.7 0.5 ± 0.7	1.0 ± 0.7 0.4 ± 0.6	1.1 ± 0.7 0.3 ± 0.5	1.3 ± 0.7 0.3 ± 0.5	1.0 ± 0.7 0.6 ± 0.6	0.201 0.053	1.1 ± 0.7 0.4 ± 0.6	0.522 **0.016**

Absolute values and relative change in disease activity composite scores and markers at 6 months of therapy in rheumatoid arthritis patients, according to biologic therapy. Continuous variables presented as mean ± standard deviation; categorical variables are expressed as number (percentage). Final number of patients is indicated where there was missing data. *P* value significant at <0.05; significant differences highlighted in bold. ^§^Comparison of groups according to biologic using ANOVA or chi-square test, as appropriate; ^¥^comparison of TNFi versus tocilizumab groups using Student's *t*-test or chi-square test, as appropriate.

**Table 3:** Logistic regression and propensity score-based analyses to predict treatment response with tocilizumab versus TNFi.

	*N*	Remission			Low disease activity		
		Odds ratio	95% CI	*P* value	Odds ratio	95% CI	*P* value
		DAS28 < 2.6			DAS28 < 3.2		
Multivariate LR	489	13.3	6.9–25.4	**<0.001**	7.3	3.9–13.6	**<0.001**
Univariate LR + PS	489	8.3	4.4–15.4	**<0.001**	5.5	2.9–10.5	**<0.001**
Multivariate LR + PS	489	11.0	5.6–21.6	**<0.001**	6.2	3.2–12.0	**<0.001**
PS matching	259	7.9	4.3–14.6	**<0.001**	5.0	2.7–9.2	**<0.001**
PS matching + multivariate LR	259	12.3	6.0–25.4	**<0.001**	7.5	3.7–15.1	**<0.001**

		CDAI ≤ 2.8			CDAI ≤ 10		
Multivariate LR	308	2.9	1.3–6.6	**0.009**	3.0	1.5–6.1	**0.002**
Univariate LR + PS	308	2.5	1.1–5.6	**0.022**	2.3	1.1–4.6	**0.019**
Multivariate LR + PS	308	2.8	1.2–6.5	**0.016**	2.6	1.3–5.5	**0.010**
PS matching	179	2.1	0.92–5.0	0.078	2.0	1.00–4.1	**0.048**
PS matching + multivariate LR	179	3.3	1.3–8.4	**0.015**	2.6	1.2–5.6	**0.017**

		SDAI ≤ 3.3			SDAI ≤ 11		
Multivariate LR	282	4.1	1.7–9.5	**0.001**	2.9	1.4–6.3	**0.005**
Univariate LR + PS	282	3.1	1.3–7.0	**0.008**	2.2	1.04–4.8	**0.038**
Multivariate LR + PS	282	3.6	1.5–8.7	**0.005**	2.5	1.1–5.5	**0.024**
PS matching	158	2.6	1.1–6.4	**0.029**	1.6	0.8–3.5	0.209
PS matching + multivariate LR	158	4.0	1.5–10.8	**0.007**	2.2	0.95–5.0	0.065

		Boolean					
Multivariate LR	442	2.1	0.91–4.8	0.083			
Univariate LR + PS	442	1.8	0.76–4.0	0.184			
Multivariate LR + PS	442	1.9	0.77–4.8	0.159			
PS matching	216	1.2	0.54–2.9	0.629			
PS matching + multivariate LR	216	1.4	0.54–3.9	0.463			

		Good EULAR			Good/moderate EULAR		
Multivariate LR	489	6.8	3.8–12.3	**<0.001**	2.5	1.1–5.9	**0.035**
Univariate LR + PS	489	6.3	3.4–11.8	**<0.001**	1.8	0.8–4.1	0.143
Multivariate LR + PS	489	6.4	3.4–12.0	**<0.001**	1.8	0.8–4.5	0.182
PS matching	259	6.2	3.3–11.6	**<0.001**	2.2	0.93–5.2	0.074
PS matching + multivariate LR	259	7.8	4.0–15.4	**<0.001**	2.4	0.98–6.1	0.056

The odds ratio and 95% confidence interval (95% CI) for the effect of tocilizumab versus TNF inhibitors (TNFi) in the considered outcomes are represented according to the statistical methodology used. Multivariate logistic regression (LR) adjusted for other significant confounders as described in Section 2, namely, age, disease duration, number of previous biologics, and baseline disease activity (DAS28 for DAS28/Boolean remission, DAS28 low disease activity (LDA), and EULAR response; CDAI for CDAI remission/LDA; SDAI for SDAI remission/LDA). Propensity scores (PS) predicting biologic class were calculated and incorporated in the analysis, via LR and/or matching (caliper 1 : 5 with replacement). *P* value significant at <0.05; significant differences highlighted in bold.

## Abbreviations

ACPA: Anti-citrullinated protein antibodies
ACR: American College of Rheumatology
CDAI: Clinical disease activity index
CI: Confidence interval
CRP: C-reactive protein
DAS28: Disease activity score-28 joints
DMARDs: Disease modifying antirheumatic drugs
ESR: Erythrocyte sedimentation rate
EULAR: European League Against Rheumatism
HAQ: Health assessment questionnaire
LDA: Low disease activity
MTX: Methotrexate
OR: Odds ratio
PGH: Patient global health
PhGA: Physician global assessment
RA: Rheumatoid arthritis
RCTs: Randomised clinical trials
Reuma.pt: Rheumatic Diseases Portuguese Register
RF: Rheumatoid factor
SDAI: Simplified disease activity index
SJC28: Swollen joint count-28 joints
TNFi: Tumour necrosis factor inhibitors
TJC28: Tender joint count-28 joints
VAS: Visual analogue scale.
